# Viruses associated with measles-like illnesses in Uganda

**DOI:** 10.1016/j.jinf.2024.106148

**Published:** 2024-05

**Authors:** Prossy Namuwulya, Shirin Ashraf, Marc Niebel, Alfred Ssekagiri, Phionah Tushabe, Proscovia Kakooza, Lily Tong, Henry Bukenya, Hanna Jerome, Chris Davis, Molly Birungi, Irene Turyahabwe, Arnold Mugaga, James Peter Eliku, Aine Francis, Lucy Nakabazzi, Fred Nsubuga, Edson Katushabe, Annet Kisakye, Immaculate Ampeire, Ann Nanteza, Pontiano Kaleebu, Barnabas Bakamutumaho, Peninah Nsamba, Anne Kazibwe, Ana da Silva Filipe, Robert Tweyongyere, Josephine Bwogi, Emma C. Thomson

**Affiliations:** a Uganda Virus Research Institute (UVRI), Entebbe, Uganda; b MRC - University of Glasgow Centre for Virus Research (CVR), Glasgow, UK; c Ministry of Health, Kampala, Uganda; d World Health Organization, Kampala, Uganda; e College of Veterinary Medicine, Animal Resources and Biosecurity, Makerere University, Kampala, Uganda; f London School of Hygiene and Tropical Medicine (LSHTM), London, UK

**Keywords:** Measles-like illness, Serum, Metagenomic next-generation sequencing, Viruses, Uganda

## Abstract

**Objectives:**

In this study, we investigated the causes of measles-like illnesses (MLI) in the Uganda national surveillance program in order to inform diagnostic assay selection and vaccination strategies.

**Methods:**

We used metagenomic next-generation sequencing (M-NGS) on the Illumina platform to identify viruses associated with MLI (defined as fever and rash in the presence of either cough, coryza or conjunctivitis) in patient samples that had tested IgM negative for measles between 2010 and 2019.

**Results:**

Viral genomes were identified in 87/271 (32%) of samples, of which 44/271 (16%) contained 12 known viral pathogens. Expected viruses included rubella, human parvovirus B19, Epstein Barr virus, human herpesvirus 6B, human cytomegalovirus, varicella zoster virus and measles virus (detected within the seronegative window-period of infection) and the blood-borne hepatitis B virus. We also detected Saffold virus, human parvovirus type 4, the human adenovirus C2 and vaccine-associated poliovirus type 1.

**Conclusions:**

The study highlights the presence of undiagnosed viruses causing MLI in Uganda, including vaccine-preventable illnesses. NGS can be used to monitor common viral infections at a population level, especially in regions where such infections are prevalent, including low and middle income countries to guide vaccination policy and optimize diagnostic assays.

## Highlights

• We investigated viral causes of measles-like illness in Uganda between 2010 and 2019.
• Expected infections with rubella virus, human parvovirus B19, Epstein Barr virus, human herpesvirus 6B, human cytomegalovirus, varicella zoster virus and measles virus were detected.
• We detected Saffold virus in two cases.
• Other viral infections for which standard diagnostic tests are not currently available included human parvovirus 4, human adenovirus C2 and vaccine-associated poliovirus.

## Introduction

Measles-like illnesses (MLI), formally defined in Uganda for surveillance purposes as the combination of fever and rash in the presence of either cough, coryza or conjunctivitis, is a common clinical syndrome in Sub-Saharan Africa. In Uganda, MLI is tracked by the Uganda Virus Research Institute (UVRI) on behalf of the Uganda Ministry of Health in order to identify cases of measles, which cause significant morbidity and mortality. While serological diagnostic tests for measles and rubella are used routinely, diagnostic tests for other viruses associated with MLI are not currently available. We aimed to identify common viruses to enhance recommendations to broaden lab-based screening algorithms in country, to improve accurate diagnosis of illnesses associated with fever and rash for informed timely management of disease and for public health measures to prevent onward transmission.^1^ MLI is known to be caused by multiple viral agents including parvovirus B19, rubella virus, human herpesviruses, adenoviruses and enteroviruses in other regions.^2^, ^3^, ^4^ Viruses of high consequence including the viral hemorrhagic fever viruses, Ebola virus and Crimean-Congo hemorrhagic fever virus (CCHFV) may also present with fever and rash and have been detected as localized outbreaks in Uganda.^5^, ^6^ In this study, we aimed to identify viruses present in the sera of individuals with MLI using unbiased M-NGS from patients that had tested negative for measles IgM, to use as the basis of recommendations on widening diagnostic assays specific to Uganda.

Metagenomic next-generation sequencing (M-NGS) is now a widely-used technology allowing for the detection of genetic signatures of pathogens in multiple specimen types.^7^ It can detect a range of pathogens, including co-infections and can yield results within hours.^8^, ^9^ While diagnosis at an individual level is expensive, it is more cost-effective when used for public health surveillance and can be employed to widen the repertoire of existing diagnostic assays. The agnostic nature of the method allows for the detection of viruses (and other pathogens) that are novel or that have not been considered by treating physicians.^10^

This study aimed to identify viruses present in the sera of individuals diagnosed with MLI in the Ugandan population using unbiased M-NGS. Identifying circulating viruses that threaten the health of the population will guide recommendations for widening diagnostic algorithms specific to the country.

## Methods

### Study samples

This was a cross-sectional study that utilized 271 randomly selected serum specimens collected during the national measles surveillance exercise between 2010 and 2019 across Uganda and archived at the UVRI-Expanded Program on Immunization (EPI) laboratory. Measles surveillance in Uganda is based on clinical symptoms of measles-like illness (MLI), defined as fever greater than 37.5°C, maculopapular rash and either cough, conjunctivitis or coryza. The study samples were those of patients with MLI which tested negative on measles-specific IgM serological testing.

### Recruitment and ethics

Permission to use anonymized specimens without written informed consent was obtained from the Uganda Ministry of Health, the UVRI research and ethics committee (Ref No. GC/127/19/09/740), and the Uganda National Council of Science and Technology (HS 2741).

### ELISA serological assays

Sera were subjected to measles and rubella IgM assays in parallel within 7 days of receipt. Samples collected between 2010 and 2017 were tested using Enzygnost (Siemens Healthcare Diagnostic Products, GmbH, Marburg, Germany) while those collected between 2018 and 2019 were tested with kits from Euroimmun (Euroimmun Medizinische Labordiagnostika AG, Lübeck, Germany) and Serion Classic (Institut Virion/Serion GmbH, Würzburg, Germany). All assays were performed according to the manufacturer’s instructions.

### RNA extraction, library preparation, and sequencing

RNA was extracted from 200 µl of sera using the Agencourt RNAdvance blood extraction kit (Beckman Coulter) following manufacturer’s instructions and involving a DNase treatment step at 37°C for 15 minutes as previously described.^1^ Extracted RNA was converted to cDNA using Superscript III (Invitrogen) and random hexamers. The cDNA was converted to double-stranded DNA using the NEBNext Ultra II Non-Directional RNA Second Strand Synthesis Module (New England Biolabs). Library preparation was performed using the KAPA LTP Library Preparation Kit from Kapa Biosystems, and the process was automated using the Biomek FXP liquid handler from Beckman Coulter. NEBNext Multiplex Oligos were utilized for indexing and amplification.^11^ The amplified libraries were quantified using a Qubit 3.0 fluorimeter from Invitrogen and a 4200 TapeStation from Agilent. The libraries were then pooled in equal molar amounts and sequenced using the NextSeq 500/550 High Output v2.5 300 cycle kit, employing a paired-end read configuration with a length of 2 × 150 bp. In order to lower the chance of cross-contamination, a stringent one-way sequencing system with separated rooms for each stages of the NGS sequencing process was followed.

### Bioinformatic analysis and quality control

Raw fastq files were analyzed with FastQC software v. 0.11.9 (http://www.bioinformatics.babraham.ac.uk/projects/fastqc).^12^ Reads were checked per base for sequence quality, GC content, duplicated sequences, length, and presence of adaptors. Quality-checked sequences were then trimmed and exported as clean FASTQ files for downstream analysis. Human genomic reads were removed by mapping to the human reference genome.^13^ Residual reads were subjected to *de novo* assembly by IDBA-UD version 0.19 and SPAdes version 3.11.1. Diamond BLASTx version 0.8.20.82 was used to identify probable viral contigs and then BLASTn version 2.7.1 was used to confirm those with an e-value of less than 1e^−5^. Reference mapping was performed for known and expected viruses using Tanoti v.1 (https://github.com/vbsreenu/Tanoti) to generate consensus sequences and record genome coverage and mapped reads for each sample (Supplementary Fig. 1).

Bioinformatic reporting was limited to (a) samples with at least 5 viral sequence reads detected and reads mapping to at least two areas of the viral genome (b) samples with >50% unique reads compared with all other samples on the same run, and (c) phylogenetically distinct sequences or sequences with read numbers >10% of any sample in the same clade (1% pairwise distance). The script used to detect identical reads from the same run is available at https://github.com/ecthomson/Contamination-Filter and the script used to identify similar reads is available at https://github.com/ecthomson/Contamination-Phylogeny. Raw mapped fastq reads were submitted to SRA under Bioproject Accession PRJNA1048868.

### Phylogenetic analysis

Consensus sequences were aligned with relevant reference sequences using MAFFT v7.313.^14^ IQTREE v1.6.12 was used to generate maximum likelihood trees based on the best model per tree using 1000 ultrafast bootstrap replicates.^15^ Generated trees were mid-point rooted or rooted with an outgroup, annotated and visualized using Figtree v.1.4.4^16^ and iTOL v6.^17^

## Results

### Demographics characteristics

**Fig. 1:**
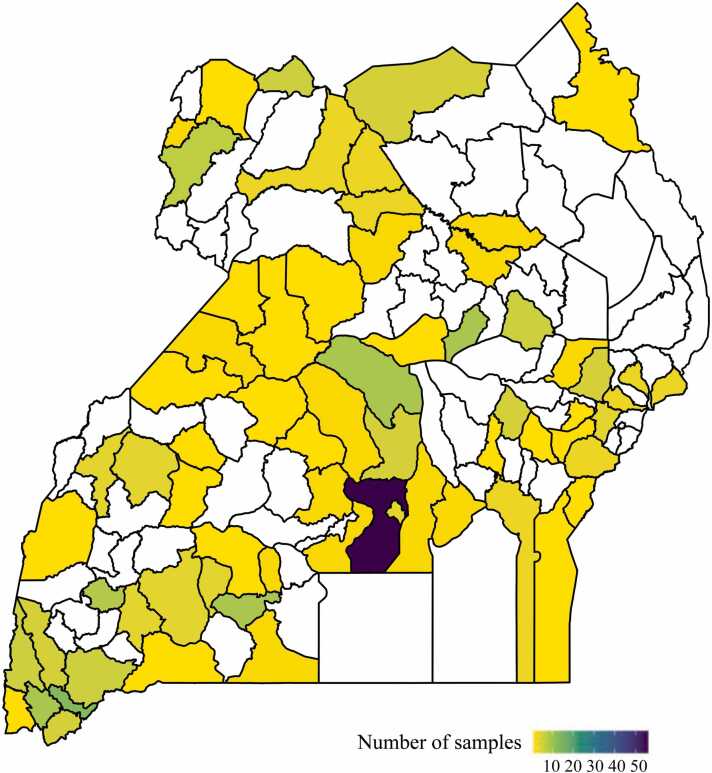
Patient demographics and sample distribution by district. The source of MLI samples from across Uganda is shown by district as a heatmap.

Two hundred and seventy-one MLI surveillance serum specimens collected between 2010 and 2019 were available for analysis. Fifty-three percent (143/271) were male and 47% (128/271) female. The median age at the time of specimen collection was 5 years (age range 0–29 years). The geographical source of available specimens is shown in Fig. 1. Distribution of samples collected across each year is summarized in Supplementary Table 1.

### Detected viruses

Following *de novo* assembly and reference mapping analysis, 32% (87/271) of specimens were found to contain viral genomes known to infect humans, of which 16% (44/271) were with known pathogens. Viral pathogens were divided into those commonly associated with MLI (Table 1) and those rarely associated with MLI (Table 2). Viruses known to infect humans but not known to be associated with human disease were present in 20% (53/271) of samples (Supplementary Table 2). Co-infecting viruses occurred in 23/271 (8.5%) of samples (Supplementary Table 3), most commonly with anelloviruses which are known to occur almost ubiquitously in human samples. Co-infection with known viral pathogens was not observed. *De novo* analysis also identified several partial viral genomes of unknown pathogenic potential (Supplementary Table 4), and a novel near full genome within the family *Hypoviridae*.

**Table 1:** Viruses commonly associated with rash and fever.

Virus	No.	Lab_ID	Districts	Year onset	Age (years)	nt coverage (%)	Mapped reads	Nearest genome	Blastn %
Rubella virus 2B (RuV)	12	MLI-UGA-20	Kyenjojo	2018	2	2436 (25%)	38	OM735672.1	96.7
		MLI-UGA-21	Kiruhura	2018	3	1129 (12%)	26	OM735663.1	97.9
		MLI-UGA-24	Tororo	2018	3	971 (10%)	26	OM735663.1	99.6
		MLI-UGA-31	Lyantonde	2018	20	4067 (42%)	61	OM735663.1	99.3
		MLI-UGA-32	Soroti	2018	10	6023 (62%)	92	OM735663.1	94.3
		MLI-UGA-38	Kumi	2018	5	1355 (14%)	16	OM735663.1	93.4
		MLI-UGA-180	Busia	2018	7	352 (4%)	8	OM735661.1	98.5
		MLI-UGA-226	Ntungamo	2018	6	560 (6%)	8	OM735672.1	99.7
		MLI-UGA-70	Kanungu	2019	5	412 (4%)	6	OM735663.1	98.6
		MLI-UGA-87	Kanungu	2019	6	808 (8%)	6	OM735663.1	99.6
		MLI-UGA-88	Kanungu	2019	6	1014 (10%)	10	OM735663.1	99.2
		MLI-UGA-93	Buliisa	2019	7	1386 (14%)	12	OM735663.1	98.3
Measles virus B3 (MV)	6	MLI-UGA-25	Mbarara	2018	3	1645 (11%)	14	MN630023.1	100
		MLI-UGA-192	Mbarara	2018	15	691 (4%)	16	MN630023.1	100
		MLI-UGA-34	Kyenjojo	2018	10	13537 (86%)	495	ON642799.1	98.4
		MLI-UGA-35	Butaleja	2018	12	1084 (7%)	12	ON642799.1	100
		MLI-UGA-39	Bukomansimbi	2018	1	15563 (99%)	589	ON642799.1	99.5
		MLI-UGA-40	Bukomansimbi	2018	8	15688 (100%)	1188	ON642799.1	99.7
Human Parvovirus B19 (B19V)	5	MLI-UGA-5	Wakiso	2013	9	5545 (97%)	520	FN598217.1	99.5
		MLI-UGA-6	Wakiso	2013	2	4736 (85%)	80	FN598217.1	98.9
		MLI-UGA-7	Wakiso	2013	5	3474 (62%)	52	FN598217.1	98.1
		MLI-UGA-10	Kampala	2013	1	5396 (96%)	664	FN598217.1	99.5
		MLI-UGA-94	Wakiso	2010	7	5589 (100%)	4575678	FN598217.1	99.6
Varicella zoster virus (VZV)	1	MLI-UGA-8	Wakiso	2013	4	29549 (24%)	435	PP169944.1	98.7
Epstein-Barr Virus (EBV)	3	MLI-UGA-9	Wakiso	2013	4	1631 (1%)	28	MK540461.1	100
		MLI-UGA-27	Nakaseke	2018	4 months	463 (0.3%)	52	OR652423.1	100
		MLI-UGA-109	Mityana	2018	1	2750 (1.6%)	61	MK973062.1	95.5
Human betaherpesvirus 6B (HHV6B)	2	MLI-UGA-13	Kampala	2014	11 months	11779 (7.3%)	155	MF511175.2	96.8
		MLI-UGA-75	Rukiga	2019	8	16741 (10%)	517	MF994829.1	100
Human cytomegalovirus (HCMV)	1	MLI-UGA-23	Sironko	2018	5	464 (0.2%)	6	KY490080.1	100

**Table 2:** Viruses less commonly associated with rash and fever.

Virus	No.	Lab_ID	Districts	Year_onset	Age (years)	nt coverage (%)	Mapped reads	Nearest genome	Blastn %
Saffold virus 3 (SAFV-3)	2	MLI-UGA-3	Wakiso	2010	4	6509 (82%)	655	HM181999.1	88.6
Saffold virus 8 (SAFV-8)		MLI-UGA-83	Bukedea	2019	11	2792 (35%)	19	AB747255.1	98
Human Parvovirus 4 (genotype 3)	5	MLI-UGA-90	Moyo	2019	2	1731 (33%)	21	KU871315.1	99.6
		MLI-UGA-91	Moyo	2019	5	986 (19%)	16	KU871315.1	98
		MLI-UGA-92	Moyo	2019	4	5197 (99%)	435778	KU871315.1	96.3
		MLI-UGA-85	Budaka	2019	6	738 (14%)	6	KU871315.1	96.6
		MLI-UGA-89	Kabarole	2019	1	399 (7%)	8	EU874248.1	96
Hepatitis B virus (HBV)	2	MLI-UGA-29	Oyam	2018	13	3212 (100%)	4681	KP168420.1	98.6
		MLI-UGA-30	Sembabule	2018	8	3159 (98%)	4961	GU563545.1	99.4
Poliovirus 1/Enterovirus C (PV)	1	MLI-UGA-65	Otuke	2019	16	882 (12%)	24	MG571844.1	96.3
Human adenovirus C2 (HAdV-C2)	4	MLI-UGA-56	Maracha	2019	4	4462 (12%)	180	OR777170.1	99.2
		MLI-UGA-59	Kapchorwa	2019	5	4806 (13%)	253	OR777170.1	99.9
		MLI-UGA-57	Kaabong	2019	3	4901 (13%)	286	MN513342.1	99.4
		MLI-UGA-118	Hoima	2010	6	400 (1%)	6	MN088492.1	93.1

**Fig. 2:**
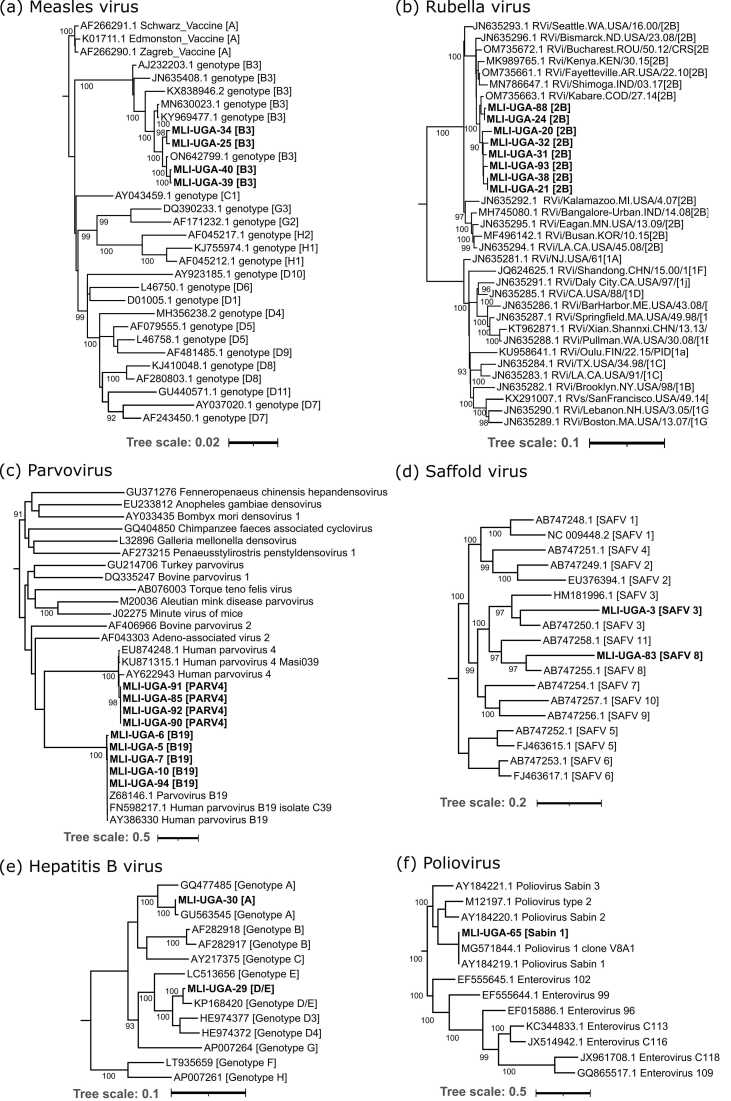
Maximum likelihood analysis of viruses detected in patients with MLI, including **(a)** Measles virus, **(b)** Rubella virus, **(c)** Parvovirus, **(d)** Saffold virus, **(e)** HBV, **(f)** Poliovirus. Bootstrap values >90 following 1000 replicates are indicated on relevant branches.

Although patients who tested IgM positive were excluded from the study, we detected measles virus genome in 6 patients aged between 1 and 15 years old (Table 1; Supplementary Table 5) in Bukomansimbi and Kyenjojo districts in 2018, following a confirmed widespread outbreak across the country between 2018–2019. These infections were noted to be clustered phylogenetically within genotype B3 (Fig. 2a).

Amongst other viruses associated with MLI, we identified rubella virus as the commonest infection. Rubella genome was detected in 12 samples from multiple areas of Uganda, in patients aged 2–20 years old. These cases occurred in 2018–2019, likely reflecting a widespread outbreak at this time. In keeping with this, a single phylogenetic cluster of genotype 2B was noted (Fig. 2b). Six of these samples also tested IgM positive for rubella (Supplementary Table 5).

We detected another common cause of childhood illness, human parvovirus B19 in 5 cases aged 1–9 years, four of which occurred in Wakiso district and one in Kampala indicating a more localized urban outbreak during 2010–2013 around the capital city (Fig. 2c).

Other detected viruses commonly associated with rash and fever in children included the herpesviruses Epstein Barr virus (EBV), cytomegalovirus (CMV), varicella zoster virus (VZV) and human herpesvirus 6 (HHV6).

Among the viruses that may more rarely be associated with febrile illness and rash, we detected human parvovirus 4 (genotype 3) (Fig. 2c), human adenovirus C2 (HAdV C2) and Saffold virus (SAFV) genotypes 3 and 8 (Fig. 2d). SAFV-3 and SAFV-8 are members of the *Cardiovirus B* species (family *Picornaviridae*). SAFV3, with 89% nucleotide homology to strains from the Netherlands and Pakistan was detected in a 4-year old male child (MLI-UGA-3) presenting with high fever, rash and cough on 10th April 2010 from Wakiso district. SAFV8, with 98% nucleotide homology to strains from Pakistan was present in a 11 year old child presenting with a rash, fever, cough and coryza from Bukedea district with rash onset on 21st April 2019 (MLI-UGA-83). The blood-borne hepatitis B virus (HBV) (Fig. 2e) was also detected and may have represented either acute or chronic infection (HBV may occasionally be associated with fever and rash during seroconversion). We detected human poliovirus (*Enterovirus C*; Fig. 2f) in 1 specimen. The blood sample was collected from a 16-year old patient who presented with cough, conjunctivitis and rash during 2019 and who had no recorded neurological symptoms. The sample aligned with the poliovirus vaccine Sabin 1 VP1 gene (accession number V01150.1) with 100% homology, indicating that the virus in our study sample was a Sabin-like poliovirus and not a vaccine-related revertant strain.

A number of viruses known to infect humans without causing human disease (Supplementary Table 2) included several circular DNA viruses in the families *Anelloviridae* (including the near-ubiquitous *Alphatorquevirus* genus),^18^ *Circoviridae* (*Cyclovirus*) and *Genomoviridae* genera. We detected 10 patient samples containing the human blood-associated dicistrovirus (KY973643.1), previously detected in human samples in Peru and in Nigeria.^19^, ^20^ We also detected the *Human Pegivirus* (HPgV) within the family *Flaviviridae* in this category.

Finally, several viruses were detected that were considered to be of uncertain significance, for which, the nearest genomes have been sequenced previously only in insect species (Supplementary Table 4). This included a full genome of 12 kb for a virus belonging to the *Hypoviridae* family with 60% amino acid identity to Wuhan insect virus-14, in an 18-year-old patient from Kabale (MLI-UGA-41) presenting with fever, rash, cough and coryzal symptoms. Phylogenetic analysis placed this genome closest to viruses obtained from ticks previously, while overall the clade contains viruses that primarily infect fungi (Supplementary Fig. 2). Further studies to characterize the pathogenicity, if any, of these viruses in human subjects with fever are indicated, including case-control sequencing cohort studies, new serological assays and where possible i*n vitro* and *in vivo* model systems.

## Discussion

This study aimed to identify viral pathogens circulating in Uganda that could be targeted for widening the diagnostic repertoire for people presenting with measles-like illness. The study utilized agnostic M-NGS of serum samples collected during national surveillance in order to identify viruses associated with MLI in the Ugandan population. Common childhood illnesses predominated, including measles, rubella, parvovirus B19, VZV, CMV and EBV. We also detected several pathogens within the family *Picornaviridae*, including poliovirus and SAFV. A small number of viruses, only previously detected in insect species were also identified and require further studies to investigate their role in human infection.

The most commonly detected viral pathogen was rubella virus (genotype 2B), an infection that is vaccine-preventable, and although associated with only mild illness in the general population, is associated with an extremely high risk (near to 100%) of congenital deafness, cataracts, cardiac defects or other severe manifestations in the first trimester of pregnancy.^21^, ^22^ These and other results^23^ indicated that widespread infection was present in Uganda during 2018–2019 and indicates a need to enhance vaccination of the Ugandan population for this infection, especially in girls and women of child-bearing age. Rubella vaccination was therefore introduced in Uganda in October 2019 and future surveillance will determine the impact of this measure. Of the 12 samples identified with rubella genomes in this study, 6 (50%) were rubella IgM negative. We therefore recommend that the addition of PCR testing for rubella virus could be considered in addition to serology to enhance surveillance and the response to the introduction of vaccination.

Although samples had been pre-screened with measles IgM testing, measles virus genome was also commonly detected and likely represents infection within the seroconversion window period or a lack of serological response to acute infection. The inclusion of routine PCR screening in addition to IgM serology in Uganda would improve the sensitivity of measles diagnosis, as recommended for this reason “where possible” by the World Health Organisation (WHO).^24^, ^25^ Ongoing measles outbreaks are of concern as there are 140,000 deaths per year from measles globally, mostly in children under 5 years of age.^26^ It is a vaccine-preventable disease, and although vaccination is widespread in Uganda, it is delivered early at the age of 9 months, and only a single dose is routinely delivered, alongside booster doses in the event of a known outbreak. If single dose vaccination is delivered earlier than 12 months of age, (usually 9 months in endemic countries), the resulting effectiveness reduces from around 92% to 85%^26^ and mean antibody titers are lower.^27^ This increases to 95% if two doses are given at around 12 months of age. Lower effectiveness in young infants may be due to the presence of maternal antibodies that neutralize the vaccine virus before an effective immune response can be generated. The timing of vaccination must be balanced by the likelihood of exposure in countries where measles is endemic and effectiveness at prevention of severe disease.^28^ A second dose of vaccine reduces the likelihood of breakthrough infection during measles outbreaks.^29^ In order to eliminate measles, the WHO recommends 2 doses of the measles containing vaccine (MCV2) to ensure immunity and prevent outbreaks. MCV2 was introduced in Uganda in October 2022. Further studies like this one will be indicated to monitor the impact of second dose vaccination.

We detected other viruses that are well-known to cause fever and rash in other settings, including human parvovirus B19, HHV6B, EBV, VZV and CMV. Human parvovirus B19 causes “fifth disease”, otherwise known as “slapped cheek syndrome” due to the appearance of a facial rash in infected individuals and more rarely causes polyarthropathy and anemia. The herpesvirus HHV6B causes roseola infantum, and EBV and CMV are commonly associated with glandular fever-like illness, characterized by rash, fever, lymphadenopathy and bilateral tonsillar exudate. The subtleties of clinical presentation of childhood infections associated with rash and fever are not well described in the Ugandan population, and characteristics of the rash e.g. macular, papular, vesicular, purpuric alongside distribution and accompanying features such as lymphadenopathy are not routinely reported or assessed during routine MLI screening in Uganda. Further clinical description and reporting could help with clinical diagnosis in the absence of resources for additional testing without additional cost.

A number of viruses less commonly associated with MLI were also detected in this study, including SAFV, poliovirus, human parvovirus 4 and HBV. The blood-borne virus HBV can cause fever and rash in the acute phase,^30^, ^31^, ^32^ and its detection in this study may represent acute infection or chronic incidental infection.

The both viruses within the family *Picornaviridae* (poliovirus (*Enterovirus C*) and SAFV) may cause a spectrum of mild to severe illness. SAFV is associated with rash and fever while rash is not well-described in poliovirus infection.^33^ Both may also cause conjunctivitis as well as more severe symptoms affecting the cardiovascular and central nervous systems, including acute flaccid paralysis. Poliovirus was a common infection prior to vaccination in Uganda and was associated with a spectrum of symptoms, most importantly paralysis and respiratory failure, in a low but significant proportion of children. Wild polioviruses have been eliminated in Uganda. However, vaccine-derived polioviruses (VDPV) that may occur following live virus vaccine, are associated with reversion of neurotropic mutations, and can cause acute flaccid paralysis.^34^ Sabin-like poliovirus, as detected in this study (poliovirus derived from vaccine) is not associated with reversion mutations or neurotropic disease.^35^ In this case, we detected a poliovirus that was identical within the Sabin 1 VP1 gene and was therefore a Sabin-like poliovirus type 1. It occurred in a 16-year old who had not recently received poliovirus vaccination and therefore is likely to have acquired the infection from a recently vaccinated contact. There was no record of neurological symptoms. Detection of virus in a 16-year-old individual indicates that coverage of vaccination may not be complete and highlights the possibility of generation of VDPV viruses due to circulation of live vaccine virus as has recently been shown in other studies in Uganda.^36^

SAFV virus was first sequenced in 2007 from a sample obtained from an 8-month old infant in 1981.^37^ We detected two cases that were highly divergent strains within the SAFV3 and SAF8 genotypes SAFV3 identified in this study came from a sample from 2010 and to our knowledge, is the oldest record of a Saffold genome from Africa, although sequences from the region have been previously published. The detection of SAFV has been associated with acute febrile illness, diarrhea, pharyngitis and meningitis in previous case series.^38^, ^39^, ^40^ It has also been detected in cases of acute flaccid paralysis although causation was not fully established in these cases.^41^, ^42^ While the strains detected in this study were highly divergent, the SAFV3 clustered with 89% nucleotide identity to variants previously described in the Netherlands and Pakistan and the SAFV8 with 98% identity to variants previously detected in Pakistan. Based on the phylogenetic analysis of the VP1 gene, 11 genotypes have been identified with SAFV2 and SAFV3 having the highest prevalence globally.^43^

Human parvovirus 4 (PARV-4) was first described in 2005 in an injecting drug user with influenza-like symptoms.^44^ Genotypes 1 and 2 have since been detected in cohorts with blood-borne viruses in North America and Europe.^45^ In Africa, infections are not as clearly associated with blood-borne infection, and are predominantly genotype 3, as in this case. Clinical presentations reported to be associated with PARV-4 include acute influenza-like illness, encephalitis, acceleration of HIV disease, and fetal abnormalities, although whether or not the virus is a pathogen or bystander in such cases is not fully understood.^45^, ^46^

Several circular DNA viruses with no known association with disease were also detected in this study.^18^ These included three families of circular DNA viruses; the families *Anelloviridae, Cycloviridae*, and *Genomoviridae*.^47^ Although our extraction process involves DNase treatment, aimed to maximize RNA recovery, the presence of DNA virus genomes can also incomplete DNA removal or an RNA stage during virus replication. We also detected Human Pegivirus (HPgV), a single-stranded positive-sense RNA virus that belongs to the *Flaviviridae* family in 9 cases. It was first identified in 1995 during the search for new hepatitis viruses but an initial association with hepatitis has never been confirmed.^48^, ^49^

In summary, M-NGS is a powerful agnostic method that facilitates the diagnosis of pathogens in clinical samples, irrespective of clinical acumen and this study has identified common circulating undiagnosed viruses associated with MLI in Uganda. It indicates that improved vaccination programs and cost-effective diagnostic methods may help to reduce the burden of disease associated with these pathogens.

There were several limitations to our study. While the detection of known pathogens is straight-forward, some detected viruses are not known to be associated with clinical disease. The true medical relevance of such viruses requires further investigation. Our study design did not allow us to identify causation associated with novel or emerging viruses. Carefully collected matched control samples would be required to attribute true association with newly detected viruses, and mechanistic studies of pathogenesis as well as evidence of serological response are indicated to investigate some of the viruses of unknown potential to cause disease detected in this study.

## Conclusion and recommendations

The data obtained in this study provide an unbiased baseline assessment of viruses associated with MLI in Uganda for clinical, public health teams and policymakers, and is of relevance to other countries in the same region. Developing or utilizing available serological and amplicon-based PCR diagnostic tests for commonly identified viruses including rubella virus, measles virus, human parvovirus B19, EBV, CMV, HHV6B, poliovirus, Saffold virus and human adenoviruses, would enable prompt identification and disease management, but must be cost-effective in this resource-constrained setting. Further research to investigate the association of disease with PARV-4, dicistroviruses and the viruses that have previously only been detected in insect species are also indicated. The detection of a transmitted Sabin-like poliovirus suggests that there is circulation of such viruses in the community and a risk of the development of revertant neurotropic viruses that cause disease, including flaccid paralysis. M-NGS has a critical future role in tracking circulating viruses in Uganda and other countries around the world. While the use of M-NGS for diagnostic purposes in individual patients is likely to be prohibitive due to the need for technical expertise and cost, regular studies carried out at a *population* level, will enable a focus on the role of viruses in key patient populations and the development of cost-effective diagnostic assays.

## Funding

This work was supported by the Medical Research Council-Centre for Virus Research (CVR) University of Glasgow (MC_UU_1201412), Wellcome (102789/Z/13/Z), a Royal Society of Tropical Medicine and Hygiene (RSTMH) small grant 2018 and GeMVi: Application of Genomics and Modelling to the Control of Virus Pathogens-National Institute for Health and Care Research (NIHR) (project reference 17/63/82) using UK aid from the UK Government to support global health research.

## Declaration of Competing Interest

The authors declare that they have no known competing financial interests or personal relationships that could have appeared to influence the work reported in this paper.

## References

[1] Jerome H., Taylor C., Sreenu V.B., Klymenko T., Filipe A.D.S., Jackson C. (2019). Metagenomic next-generation sequencing aids the diagnosis of viral infections in febrile returning travellers. J Infect.

[2] Wawina T.B., Tshiani O.M., Ahuka S.M., Pukuta E.S., Aloni M.N., Kasanga C.J. (2017). Detection of human parvovirus B19 in serum samples from children under 5 years of age with rash–fever illnesses in the Democratic Republic of the Congo. Int J Infect Dis.

[3] Li X., Lin Z., Liu J., Tang Y., Yuan X., Li N. (2020). Overall prevalence of human parvovirus B19 among blood donors in mainland China: a PRISMA-compliant meta-analysis. Medicine.

[4] de Moraes J.C., Toscano C.M., de Barros E.N.C., Kemp B., Lievano F., Jacobson S. (2011). Etiologies of rash and fever illnesses in Campinas, Brazil. J Infect Dis.

[5] Atim S.A., Niebel M., Ashraf S., Vudriko P., Odongo S., Balinandi S. (2023). Prevalence of Crimean-Congo haemorrhagic fever in livestock following a confirmed human case in Lyantonde district, Uganda. Parasites Vectors.

[6] Nyakarahuka L., Mulei S., Whitmer S., Jackson K., Tumusiime A., Schuh A. (2022). First laboratory confirmation and sequencing of Zaire ebolavirus in Uganda following two independent introductions of cases from the 10th Ebola Outbreak in the Democratic Republic of the Congo, June 2019. PLoS Negl Trop Dis.

[7] Diao Z., Han D., Zhang R., Li J. (2022). Metagenomics next-generation sequencing tests take the stage in the diagnosis of lower respiratory tract infections. J Adv Res.

[8] Malla M.A., Dubey A., Kumar A., Yadav S., Hashem A., Allah E.F.A. (2019). Exploring the human microbiome: the potential future role of next-generation sequencing in disease diagnosis and treatment. Front Immunol Front Media S A.

[9] Zhong Y., Xu F., Wu J., Schubert J., Li M.M. (2020). Application of next generation sequencing in laboratory medicine. Ann Lab Med.

[10] Kha Tu N.T., Thu Hong N.T., Han Ny N.T., Phuc T.M., Thanh Tam P.T., van Doorn H.R. (2020). The virome of acute respiratory diseases in individuals at risk of zoonotic infections. Viruses.

[11] Tong L. Discovery of RNA and DNA viruses using next generation sequencing: Metagenomics V.1; 2023.

[12] Andrews S. A quality control tool for high throughput sequence data. Babraham Bioinformatics; 2010 [updated 01-03-23]. Available from: https://www.bioinformatics.babraham.ac.uk/projects/fastqc/. Accessed: 01/02/2024.

[13] Ruffalo M., LaFramboise T., Koyutürk M. (2011). Comparative analysis of algorithms for next-generation sequencing read alignment. Bioinformatics.

[14] Katoh K., Rozewicki J., Yamada K.D. (2019). MAFFT online service: multiple sequence alignment, interactive sequence choice and visualization. Brief Bioinform.

[15] Nguyen L.T., Schmidt H.A., von Haeseler A., Minh B.Q. (2015). IQ-TREE: a fast and effective stochastic algorithm for estimating maximum-likelihood phylogenies. Mol Biol Evol.

[16] Chen S.C., Ogata A. (2015). MixtureTree annotator: a program for automatic colorization and visual annotation of MixtureTree. PLoS One.

[17] Letunic I., Bork P. (2019). Interactive Tree Of Life (iTOL) v4: recent updates and new developments. Nucleic Acids Res.

[18] de Villiers E.M., zur Hausen H. (2009). TT viruses--the still elusive human pathogens. Pref Curr Top Microbiol Immunol.

[19] Oguzie J.U., Petros B.A., Oluniyi P.E., Mehta S.B., Eromon P.E., Nair P. (2023). Metagenomic surveillance uncovers diverse and novel viral taxa in febrile patients from Nigeria. Nat Commun.

[20] Phan T.G., Del Valle Mendoza J., Sadeghi M., Altan E., Deng X., Delwart E. (2018). Sera of Peruvians with fever of unknown origins include viral nucleic acids from non-vertebrate hosts. Virus Genes.

[21] Gregg N.M. (1991). Congenital cataract following German measles in the mother. Epidemiol Infect.

[22] Wesselhoeft Conrad M.D. (1947). Rubella (German measles). N Engl J Med.

[23] WHO. Statement from Uganda’s Minister of Health on the National Measles-Rubella and Polio Immunisation Campaign 2019. Available from: https://wwwafrowhoint/news/statement-ugandas-minister-health-national-measles-rubella-and-polio-immunisation-campaign2019. Accessed: 01/02/2024.

[24] Mulders M. Manual for the laboratory-based surveillance of measles, rubella, and congenital rubella syndrome 2018. Available from: https://www.technet-21.org/en/manual-introduction. Accessed: 01/02/2024.

[25] Hübschen J.M., Bork S.M., Brown K.E., Mankertz A., Santibanez S., Ben Mamou M. (2017). Challenges of measles and rubella laboratory diagnostic in the era of elimination. Clin Microbiol Infect.

[26] WHO. More than 140,000 die from measles as cases surge worldwide 2019. Available from: https://www.who.int/news/item/05–12-2019-more-than-140–000-die-from-measles-as-cases-surge-worldwide. Accessed: 01/02/2024.

[27] Orenstein W.A., Bernier R.H., Dondero T.J., Hinman A.R., Marks J.S., Bart K.J. (1985). Field evaluation of vaccine efficacy. Bull World Health Organ.

[28] Nic Lochlainn L.M., de Gier B., van der Maas N., van Binnendijk R., Strebel P.M., Goodman T. (2019). Effect of measles vaccination in infants younger than 9 months on the immune response to subsequent measles vaccine doses: a systematic review and meta-analysis. Lancet Infect Dis.

[29] Wichmann O., Hellenbrand W., Sagebiel D., Santibanez S., Ahlemeyer G., Vogt G. (2007). Large measles outbreak at a German public school, 2006. Pediatr Infect Dis J.

[30] Chu C., Selwyn P.A. (2010). Diagnosis and initial management of acute HIV infection. Am Fam Physician.

[31] Chen X., Fu C., Liu J., Shan L., Liu C. (2015). Recent epidemiological and clinical features of acute hepatitis B in a single center of China. Int J Clin Exp Med.

[32] Kafeero H.M., Ndagire D., Ocama P., Kato C.D., Wampande E., Kajumbula H. (2022). Disproportionate distribution of HBV genotypes A and D and the recombinant genotype D/E in the high and low HBV endemic regions of Uganda: a wake-up call for regional specific HBV management. Int J Hepatol.

[33] Huang Y.-C., Chu Y.-H., Yen T.-Y., Huang W.-C., Huang L.-M., Cheng A.-L. (2013). Clinical features and phylogenetic analysis of Coxsackievirus A9 in Northern Taiwan in 2011. BMC Infect Dis.

[34] Bouchard M.J., Lam D.H., Racaniello V.R. (1995). Determinants of attenuation and temperature sensitivity in the type 1 poliovirus Sabin vaccine. J Virol.

[35] Yeh M.T., Smith M., Carlyle S., Konopka-Anstadt J.L., Burns C.C., Konz J. (2023). Genetic stabilization of attenuated oral vaccines against poliovirus types 1 and 3. Nature.

[36] Nanteza M.B., Bakamutumaho B., Tushabe P., Namuwulya P., Birungi M., Dhatemwa R. (2023). Sabin polio virus protein 1 (VP1) evolution in patients with acute flaccid paralysis from 2010 to 2016 in Uganda. Virol J.

[37] Jones M.S., Lukashov V.V., Ganac R.D., Schnurr D.P. (2007). Discovery of a novel human picornavirus in a stool sample from a pediatric patient presenting with fever of unknown origin. J Clin Microbiol.

[38] Ugai S., Iwaya A., Taneichi H., Hirokawa C., Aizawa Y., Hatakeyama S. (2019). Clinical characteristics of saffold virus infection in children. Pediatr Infect Dis J.

[39] Cordey S., Laubscher F., Hartley M.A., Junier T., Keitel K., Docquier M. (2021). Blood virosphere in febrile Tanzanian children. Emerg Microbes Infect.

[40] Ramesh A., Nakielny S., Hsu J., Kyohere M., Byaruhanga O., de Bourcy C. (2019). Metagenomic next-generation sequencing of samples from pediatric febrile illness in Tororo, Uganda. PLoS One.

[41] Leguia M., Loyola S., Rios J., Juarez D., Guevara C., Silva M. (2015). Full genomic characterization of a saffold virus isolated in Peru. Pathogens.

[42] Mullapudi E., Nováček J., Pálková L., Kulich P., Lindberg A.M., van Kuppeveld F.J. (2016). Structure and genome release mechanism of the human cardiovirus saffold virus 3. J Virol.

[43] Zhang X.A., Lu Q.B., Wo Y., Zhao J., Huang D.D., Guo C.T. (2015). Prevalence and genetic characteristics of Saffold cardiovirus in China from 2009 to 2012. Sci Rep.

[44] Jones M.S., Kapoor A., Lukashov V.V., Simmonds P., Hecht F., Delwart E. (2005). New DNA viruses identified in patients with acute viral infection syndrome. J Virol.

[45] Prakash S., Shukla S., Bhagat A.K., Mishra H., Vangala R., Jain A. (2021). Human parvovirus 4: an emerging etiological agent in cases presenting with influenza like illness. J Med Virol.

[46] Matthews P.C., Sharp C., Simmonds P., Klenerman P. (2017). Human parvovirus 4 'PARV4' remains elusive despite a decade of study. F1000Res.

[47] Sauvage V., Foulongne V., Cheval J., Ar Gouilh M., Pariente K., Dereure O. (2011). Human polyomavirus related to African green monkey lymphotropic polyomavirus. Emerg Infect Dis.

[48] Yu Y., Wan Z., Wang J.H., Yang X., Zhang C. (2022). Review of human pegivirus: prevalence, transmission, pathogenesis, and clinical implication. Virulence.

[49] Simons J.N., Leary T.P., Dawson G.J., Pilot-Matias T.J., Muerhoff A.S., Schlauder G.G. (1995). Isolation of novel virus-like sequences associated with human hepatitis. Nat Med.

