# Supplementary material for: Viruses associated with measles-like illnesses in Uganda
Source: J Infect. 2024 May;88(5):None. doi: 10.1016/j.jinf.2024.106148 (PMC11060986; doi:10.1016/j.jinf.2024.106148)

**Supplementary Table 1: Demographics of the MLI cohort**

|  |  | **Count** | **%** |
| --- | --- | --- | --- |
| **Sex** | Male | 143 | 52.8 |
|  | Female | 128 | 47.2 |
| **Age** | <1 year | 20 | 7.4 |
|  | 1-5 years | 137 | 50.6 |
|  | 6-10 years | 80 | 29.5 |
|  | 11-15 years | 23 | 8.5 |
|  | 16-20 years | 9 | 3.3 |
|  | >20 years | 2 | 0.7 |
| **Vaccination status** | Vaccinated | 60 | 22.1 |
|  | No information | 211 | 77.9 |
| **Habitation** | Urban | 4 | 1.5 |
|  | Rural | 257 | 94.8 |
|  | Missing | 10 | 3.7 |
| **Year of collection** | 2010 | 15 | 5.5 |
|  | 2013 | 21 | 7.7 |
|  | 2014 | 24 | 8.9 |
|  | 2015 | 7 | 2.6 |
|  | 2017 | 4 | 1.5 |
|  | 2018 | 104 | 38.4 |
|  | 2019 | 96 | 35.4 |

**Supplementary Table 2: Human viruses not known to be pathogens**

| **Virus** | **No.** | **Lab_ID** | **Districts** | **Year** | **Age (years)** | **nt coverage (%)** | **Mapped reads** | **Nearest genome** | **Blastn %** |
| --- | --- | --- | --- | --- | --- | --- | --- | --- | --- |
| Torque teno midi virus | 28 | MLI-UGA-1 | Wakiso | 2010 | 4 | 183 (7%) | 64 | MZ824959.1 | 100 |
|  |  | MLI-UGA-2 | Nakasongola | 2010 | 9 | 184 (7%) | 5 | MN778130.1 | 100 |
|  |  | MLI-UGA-118 | Hoima | 2010 | 6 | 185 (7%) | 20 | MN775735.1 | 100 |
|  |  | MLI-UGA-9 | Wakiso | 2013 | 4 | 183 (7%) | 6 | MZ824959.1 | 100 |
|  |  | MLI-UGA-132 | Wakiso | 2013 | 3 | 183 (7%) | 42 | MN780418.1 | 98 |
|  |  | MLI-UGA-138 | Wakiso | 2014 | 3 | 183 (7%) | 15 | MZ286026.1 | 100 |
|  |  | MLI-UGA-139 | Wakiso | 2014 | 2 | 184 (7%) | 40 | MN779003.1 | 99.5 |
|  |  | MLI-UGA-108 | Wakiso | 2014 | 10 months | 312 (12%) | 34 | MN775735.1 | 99.5 |
|  |  | MLI-UGA-142 | Wakiso | 2014 | 7 months | 180 (7%) | 22 | MZ286095.1 | 98 |
|  |  | MLI-UGA-144 | Wakiso | 2014 | 5 | 183 (7%) | 17 | MN780410.1 | 99.5 |
|  |  | MLI-UGA-145 | Wakiso | 2014 | 1 | 183 (7%) | 6 | MN780402.1 | 99.5 |
|  |  | MLI-UGA-149 | Wakiso | 2014 | 2 | 119 (5%) | 20 | MN780331.1 | 99.2 |
|  |  | MLI-UGA-156 | Wakiso | 2015 | 1 | 183 (7%) | 7 | MN778744.1 | 100 |
|  |  | MLI-UGA-157 | Kasese | 2015 | 3 | 183 (7%) | 5 | MN778884.1 | 99.5 |
|  |  | MLI-UGA-158 | Wakiso | 2015 | 2 | 183 (7%) | 14 | MN778130.1 | 100 |
|  |  | MLI-UGA-14 | Buhweju | 2017 | 3 | 146 (6%) | 18 | MN780421.1 | 100 |
|  |  | MLI-UGA-161 | Buhweju | 2017 | 3 | 183 (7%) | 6 | MZ824827.1 | 99.5 |
|  |  | MLI-UGA-15 | Buhweju | 2017 | 14 | 186 (7%) | 197 | MN778070.1 | 100 |
|  |  | MLI-UGA-98 | Buhweju | 2017 | 1 | 176 (7%) | 37 | MZ286026.1 | 100 |
|  |  | MLI-UGA-26 | Mayuge | 2018 | 1 | 164 (6%) | 6 | MN780378.1 | 100 |
|  |  | MLI-UGA-43 | Kiruhura | 2019 | 2 | 193 (7%) | 8 | MN779806.1 | 98.9 |
|  |  | MLI-UGA-44 | Kaberamaido | 2019 | 5 months | 262 (10%) | 14 | MZ824771.1 | 98.9 |
|  |  | MLI-UGA-51 | Kaberamaido | 2019 | 6 | 177 (7%) | 5 | MN780326.1 | 99.1 |
|  |  | MLI-UGA-53 | Kaberamaido | 2019 | 5 | 360 (14%) | 7 | MN779155.1 | 97.7 |
|  |  | MLI-UGA-57 | Rukungiri | 2019 | 13 | 183 (7%) | 13 | MN777956.1 | 100 |
|  |  | MLI-UGA-72 | Nakasongola | 2019 | 4 | 254 (10%) | 10 | MZ824771.1 | 97.4 |
|  |  | MLI-UGA-107 | Rukiga | 2019 | 2 | 939 (36%) | 255 | MN779788.1 | 95.4 |
|  |  | MLI-UGA-77 | Rukiga | 2019 | 6 | 166 (6%) | 9 | MZ824959.1 | 99.4 |
| TTV-like mini virus | 8 | MLI-UGA-118 | Hoima | 2018 | 6 | 99 (4%) | 6 | MN770396.1 | 98.1 |
|  |  | MLI-UGA-108 | Wakiso | 2018 | 10 months | 116 (4%) | 8 | MN770631.1 | 95.7 |
|  |  | MLI-UGA-156 | Wakiso | 2019 | 1 | 102 (4%) | 8 | MN773605.1 | 97.1 |
|  |  | MLI-UGA-53 | Kaberamaido | 2019 | 5 | 1604 (57%) | 21 | MN771907.1 | 92.7 |
|  |  | MLI-UGA-100 | Rubanda | 2019 | 4 | 71 (3%) | 6 | MN773472.1 | 93.8 |
|  |  | MLI-UGA-132 | Wakiso | 2013 | 3 | 102 (4%) | 16 | MN774019.1 | 100 |
|  |  | MLI-UGA-248 | Kapchorwa | 2019 | 3 | 98 (4%) | 6 | MN774891.1 | 100 |
|  |  | MLI-UGA-44 | Kaberamaido | 2019 | 5 months | 102 (4%) | 7 | MN770077.1 | 97.6 |
| Torque teno mini virus | 2 | MLI-UGA-72 | Nakasongola | 2019 | 4 | 100 (4%) | 5 | MN768833.1 | 100 |
|  |  | MLI-UGA-55 | Moyo | 2019 | 10 | 1364 (51%) | 152 | MN768948.1 | 96.8 |
| Torque teno virus | 4 | MLI-UGA-1 | Wakiso | 2018 | 4 | 1011 (33%) | 34 | MN768003.1 | 95.7 |
|  |  | MLI-UGA-97 | Wakiso | 2019 | 18 | 1141 (37%) | 11 | MN768080.1 | 99.1 |
|  |  | MLI-UGA-98 | Buhweju | 2019 | 2 | 1357 (44%) | 158 | MN768007.1 | 99.9 |
|  |  | MLI-UGA-108 | Wakiso | 2019 | 10 months | 291 (9%) | 20 | MN768087.1 | 100 |
| Small anellovirus | 1 | MLI-UGA-66 | Kikuube | 2019 | 6 | 236 (9%) | 5 | NC_007014.1 | 90.6 |
| Cyclovirus | 2 | MLI-UGA-11 | Wakiso | 2013 | 1 | 970 (52%) | 202 | AB937980.1 | 90.3 |
|  |  | MLI-UGA-72 | Nakasongola | 2019 | 4 | 520 (28%) | 5 | KM392285.1 | 94.9 |
| Genomoviridae sp. | 5 | MLI-UGA-4 | Wakiso | 2013 | 2 | 2199 (100%) | 578 | MT309880.1 | 83.8 |
|  |  | MLI-UGA-23 | Sironko | 2018 | 1 | 547 (25%) | 8 | MT309880.1 | 95.4 |
|  |  | MLI-UGA-15 | Buhweju | 2017 | 14 | 2186 (99%) | 471 | MT309880.1 | 83.4 |
|  |  | MLI-UGA-95 | Mbarara | 2018 | 12 | 1779 (81%) | 64 | MT309880.1 | 94.2 |
|  |  | MLI-UGA-109 | Mityana | 2018 | 1 | 387 (18%) | 5 | MT309880.1 | 84.7 |
| Human blood-associated dicistrovirus | 10 | MLI-UGA-81 | Nakasongola | 2019 | 4 | 9566 (100%) | 334435 | KY973643.1 | 95 |
|  |  | MLI-UGA-99 | Wakiso | 2014 | 3 | 8816 (92%) | 929 | KY973643.1 | 94.7 |
|  |  | MLI-UGA-100 | Rubanda | 2019 | 4 | 9566 (100%) | 2428 | KY973643.1 | 95.4 |
|  |  | MLI-UGA-92 | Moyo | 2019 | 4 | 9566 (100%) | 1677 | KY973643.1 | 95.1 |
|  |  | MLI-UGA-101 | Nakasongola | 2019 | 9 | 9566 (100%) | 16132 | KY973643.1 | 95.2 |
|  |  | MLI-UGA-21 | Kiruhura | 2018 | 3 | 1197 (13%) | 22 | OR031233.1 | 94.5 |
|  |  | MLI-UGA-43 | Kiruhura | 2019 | 2 | 1752 (18%) | 24 | OQ835731.1 | 95.9 |
|  |  | MLI-UGA-66 | Kikuube | 2019 | 6 | 1822 (19%) | 22 | KY973643.1 | 97.4 |
|  |  | MLI-UGA-107 | Rukiga | 2019 | 2 | 2009 (21%) | 29 | KY973643.1 | 94.8 |
|  |  | MLI-UGA-39 | Bukomansimbi | 2018 | 1 | 825 (9%) | 10 | OQ835731.1 | 95.7 |
| Human Pegivirus (HPgV) | 9 | MLI-UGA-12 | Kalungu | 2014 | 6 | 9299 (100%) | 41810 | OQ791475.1 | 92.4 |
|  |  | MLI-UGA-22 | Lamwo | 2018 | 5 | 5283 (57%) | 112 | KC618398.1 | 91.2 |
|  |  | MLI-UGA-31 | Lyantonde | 2018 | 20 | 1459 (16%) | 14 | LT009490.1 | 85.2 |
|  |  | MLI-UGA-33 | Soroti | 2018 | 13 | 3917 (42%) | 67 | MN551063.1 | 92.3 |
|  |  | MLI-UGA-37 | Buwheju | 2018 | 3 | 528 (6%) | 6 | KC618401.1 | 97.3 |
|  |  | MLI-UGA-47 | Amuru | 2019 | 2 | 6393 (69%) | 186 | KC618398.1 | 92.7 |
|  |  | MLI-UGA-60 | Buikwe | 2019 | 6 | 8989 (96%) | 1290 | LT009490.1 | 91.9 |
|  |  | MLI-UGA-75 | Rukiga | 2019 | 6 | 7328 (79%) | 252 | LT009490.1 | 94 |
|  |  | MLI-UGA-96 | Kanungu | 2019 | 6 | 2306 (25%) | 20 | KM670096.1 | 97.8 |

**Supplementary Table 3: Virus co-infections in study population**

| **Sample number** | **Human pathogen** | **Human virus** |
| --- | --- | --- |
| MLI-UGA-1 |  | TTMdV, TTV |
| MLI-UGA-100 |  | TTV-like mini virus, Dicistrovirus |
| MLI-UGA-107 |  | TTMdV, Dicistrovirus |
| MLI-UGA-108 |  | TTMdV, TTV, TTV-like mini virus |
| MLI-UGA-109 | EBV | Genomoviridae sp. |
| MLI-UGA-118 |  | TTMdV, TTV-like mini virus |
| MLI-UGA-132 |  | TTMdV, TTV-like mini virus |
| MLI-UGA-15 |  | TTMdV, Genomoviridae sp. |
| MLI-UGA-156 |  | TTMdV, TTV-like mini virus |
| MLI-UGA-21 | Rubella virus | Dicistrovirus |
| MLI-UGA-23 | HCMV | Genomoviridae sp. |
| MLI-UGA-31 | Rubella virus | HPgV |
| MLI-UGA-39 | Measles virus | Dicistrovirus |
| MLI-UGA-43 |  | TTMdV, Dicistrovirus |
| MLI-UGA-44 |  | TTMdV, TTV-like mini virus |
| MLI-UGA-53 |  | TTMdV, TTV-like mini virus |
| MLI-UGA-57 | HAdV-C2 | TTMdV |
| MLI-UGA-66 |  | Small anellovirus, Dicistrovirus |
| MLI-UGA-72 |  | TTMV, TTMdV, Cyclovirus |
| MLI-UGA-75 | HHV6B | HPgV |
| MLI-UGA-9 | EBV | TTMdV |
| MLI-UGA-92 | Human parvovirus 4 | Dicistrovirus |
| MLI-UGA-98 |  | TTMdV, TTV |

**Supplementary Table 4: Viruses of uncertain significance**

| **Lab_ID** | **Districts** | **Year** | **Age (years)** | **Contig size** | **Nearest accession no.** | **Nearest virus name** | **Source of nearest virus** | **BLASTn identity** |
| --- | --- | --- | --- | --- | --- | --- | --- | --- |
| MLI-UGA-84 | Bukedea | 2019 | 3 | 239 | KX883110.1 | Hubei noda-like virus 18 | Roundworm (China, 2014) | 60% |
| MLI-UGA-70 | Kanungu | 2019 | 5 | 292 | KX883955.1 | Hubei picorna-like virus 41 | Dragonfly (China, 2013) | 30% |
| MLI-UGA-81 | Nakasongola | 2019 | 4 | 3517 | KX883955.1 | Hubei picorna-like virus 41 |  | 30% |
| MLI-UGA-83 | Bukedea | 2019 | 11 | 4952 | KX883955.1 | Hubei picorna-like virus 41 |  | 30% |
| MLI-UGA-45 | Kaberamaido | 2019 | 7 months | 865 | MT153501.1 | Blattodean phasma-related virus | *Supella longipalpa* cockroach (Japan, 2011) | 99.50% |
| MLI-UGA-52 | Kaberamaido | 2019 | 5 | 495 | MT482496.1 | PNG bee virus 14 | *Apis mellifera* western honeybee (PNG, 2018) | 54% |
| MLI-UGA-67 | Bukwo | 2019 | 6 | 363 | MT482496.1 | PNG bee virus 14 |  | 54% |
| MLI-UGA-74 | Rukiga | 2019 | 5 | 801 | MT482496.1 | PNG bee virus 14 |  | 54% |
| MLI-UGA-82 | Bukedea | 2019 | 9 | 910 | MT482496.1 | PNG bee virus 14 |  | 54% |
| MLI-UGA-84 | Bukedea | 2019 | 3 | 440 | MT482496.1 | PNG bee virus 14 |  | 54% |
| MLI-UGA-40 | Bukomansimbi | 2018 | 8 | 249 | MT568536.1 | Pyongtaek Culex Ribovirus | *Culex bitaeniorhynchus* (South Korea, 2018) | 99% |
| MLI-UGA-80 | Lwengo | 2019 | 4 | 231 | MT568536.1 | Pyongtaek Culex Ribovirus |  | 99% |
| MLI-UGA-22 | Lamwo | 2018 | 5 | 541 | MH727529.1 | Solenopsis invicta virus 12 | *Solenopsis invicta* fire ants (Argentina, 2014) | 92% |
| MLI-UGA-77 | Rukiga | 2019 | 6 | 269 | MH714708.1 | Solenopsis invicta virus 6 |  | 90% |
| MLI-UGA-2 | Nakasongola | 2010 | 9 | 1194 | MH727526.1 | Solenopsis invicta virus 9 |  | 41% |
| MLI-UGA-6 | Wakiso | 2013 | 2 | 361 | LC496784.1 | Tesano Aedes virus | *Aedes aegypti* (Ghana, 2016) | 62% |
| MLI-UGA-41 | Kabale | 2018 | 18 | 12621 | KX883007.1 | Wuhan insect virus 14 | Fleas and ants (China, 2013) | 60% |
| MLI-UGA-42 | Ntungamo | 2018 | 6 | 2799 | KX883007.1 | Wuhan insect virus 14 |  | 60% |
| MLI-UGA-68 | Bukwo | 2019 | 6 | 4589 | KX883007.1 | Wuhan insect virus 14 |  | 60% |

**Supplementary Table 5: Measles and rubella viruses showing dates of rash onset and specimen collection**

| **Virus** | **No.** | **Lab_ID** | **Districts** | **Year onset** | **Last date of vaccination** | **Date onset** | **Date specimen collection** | **IgM result** | **Age (years)** |
| --- | --- | --- | --- | --- | --- | --- | --- | --- | --- |
| Rubella virus 2B (RuV) | 12 | MLI-UGA-20 | Kyenjojo | 2018 | - | 19/03/2018 | 21/03/2018 | + | 2 |
|  |  | MLI-UGA-21 | Kiruhura | 2018 | - | 20/03/2018 | 21/03/2018 | + | 3 |
|  |  | MLI-UGA-24 | Tororo | 2018 | - | 17/04/2018 | 18/04/2018 | - | 3 |
|  |  | MLI-UGA-31 | Lyantonde | 2018 | - | 23/07/2018 | 24/07/2018 | - | 20 |
|  |  | MLI-UGA-32 | Soroti | 2018 | - | 21/07/2018 | 26/07/2018 | - | 10 |
|  |  | MLI-UGA-38 | Kumi | 2018 | - | 18/11/2018 | 20/11/2018 | + | 5 |
|  |  | MLI-UGA-180 | Busia | 2018 | - | 13/03/2018 | 15/03/2018 | + | 7 |
|  |  | MLI-UGA-226 | Ntungamo | 2018 | - | 02/12/2018 | 04/12/2018 | + | 6 |
|  |  | MLI-UGA-70 | Kanungu | 2019 | - | 02/04/2019 | 03/04/2019 | +/- | 5 |
|  |  | MLI-UGA-87 | Kanungu | 2019 | - | 15/03/2019 | 16/03/2019 | + | 6 |
|  |  | MLI-UGA-88 | Kanungu | 2019 | - | 03/03/2019 | 19/03/2019 | - | 6 |
|  |  | MLI-UGA-93 | Buliisa | 2019 | 30/04/2013 | 09/04/2019 | 10/04/2019 | - | 7 |
| Measles virus B3 (MV) | 6 | MLI-UGA-25 | Mbarara | 2018 | 10/02/2016 | 28/02/2018 | 30/04/2018 | - | 3 |
|  |  | MLI-UGA-192 | Mbarara | 2018 | - | 28/02/2018 | 30/04/2018 | - | 15 |
|  |  | MLI-UGA-34 | Kyenjojo | 2018 | - | 10/10/2018 | 11/10/2018 | - | 10 |
|  |  | MLI-UGA-35 | Butaleja | 2018 | - | 20/10/2018 | 24/10/2018 | - | 12 |
|  |  | MLI-UGA-39 | Bukomansimbi | 2018 | - | 30/11/2018 | 03/12/2018 | - | 1 |
|  |  | MLI-UGA-40 | Bukomansimbi | 2018 | - | 28/11/2018 | 03/12/2018 | - | 8 |

**Supplementary Figure 1: Bioinformatic reporting algorithm:** Fastq reads were assembled using spades and IDBA software. Contigs were filtered for viral genomes using diamond blastx software against the full nr database. Reads identified as single or non-viral reads were discarded. BLASTn was then used to confirm the identity of viruses with a BLAST e score cutoff of 10^-5^. Read mapping was carried out for all samples against all detected viral genomes.


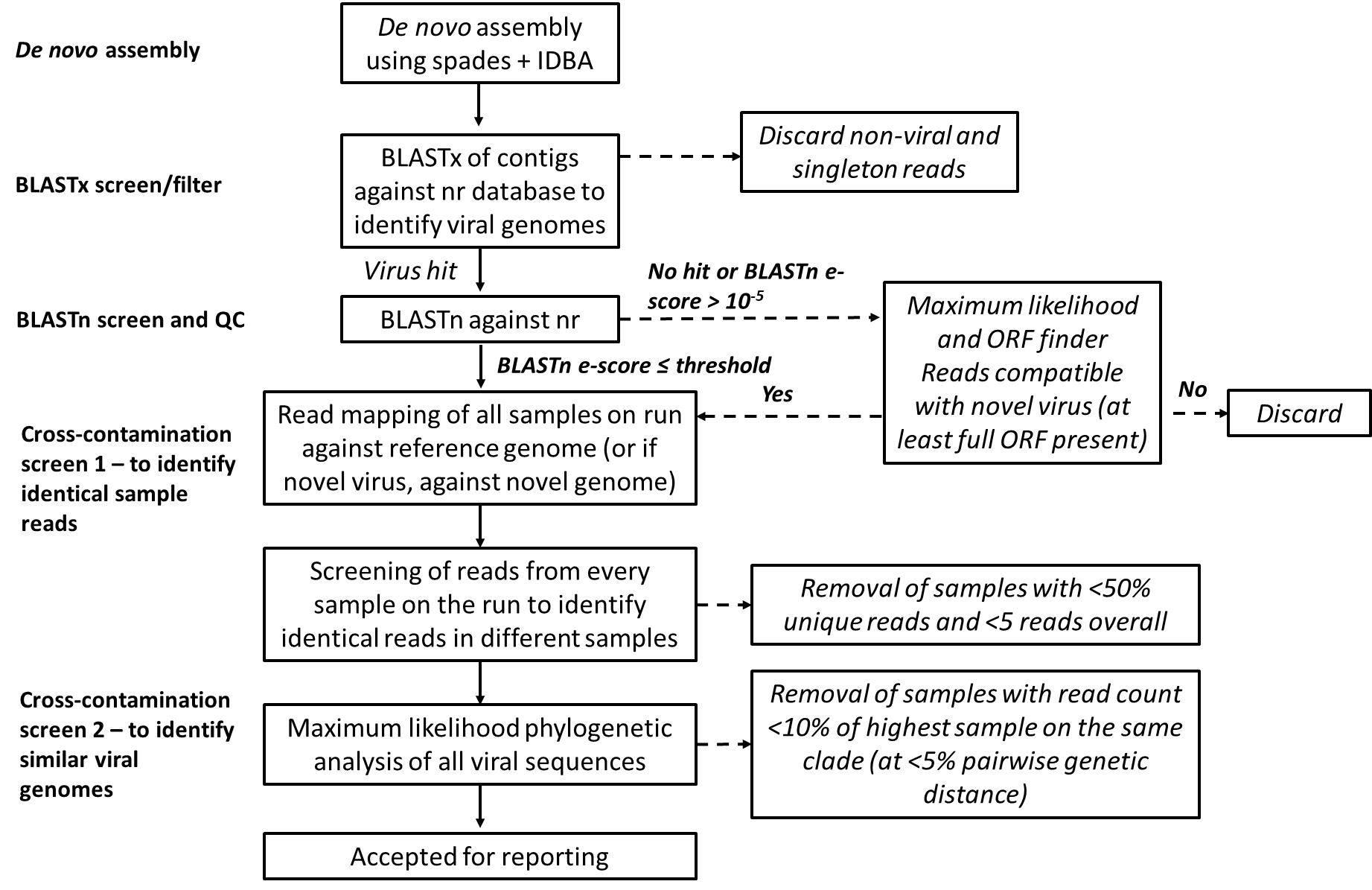


**Supplementary Figure 2: Maximum likelihood tree for novel *Hypoviridae sp*.**


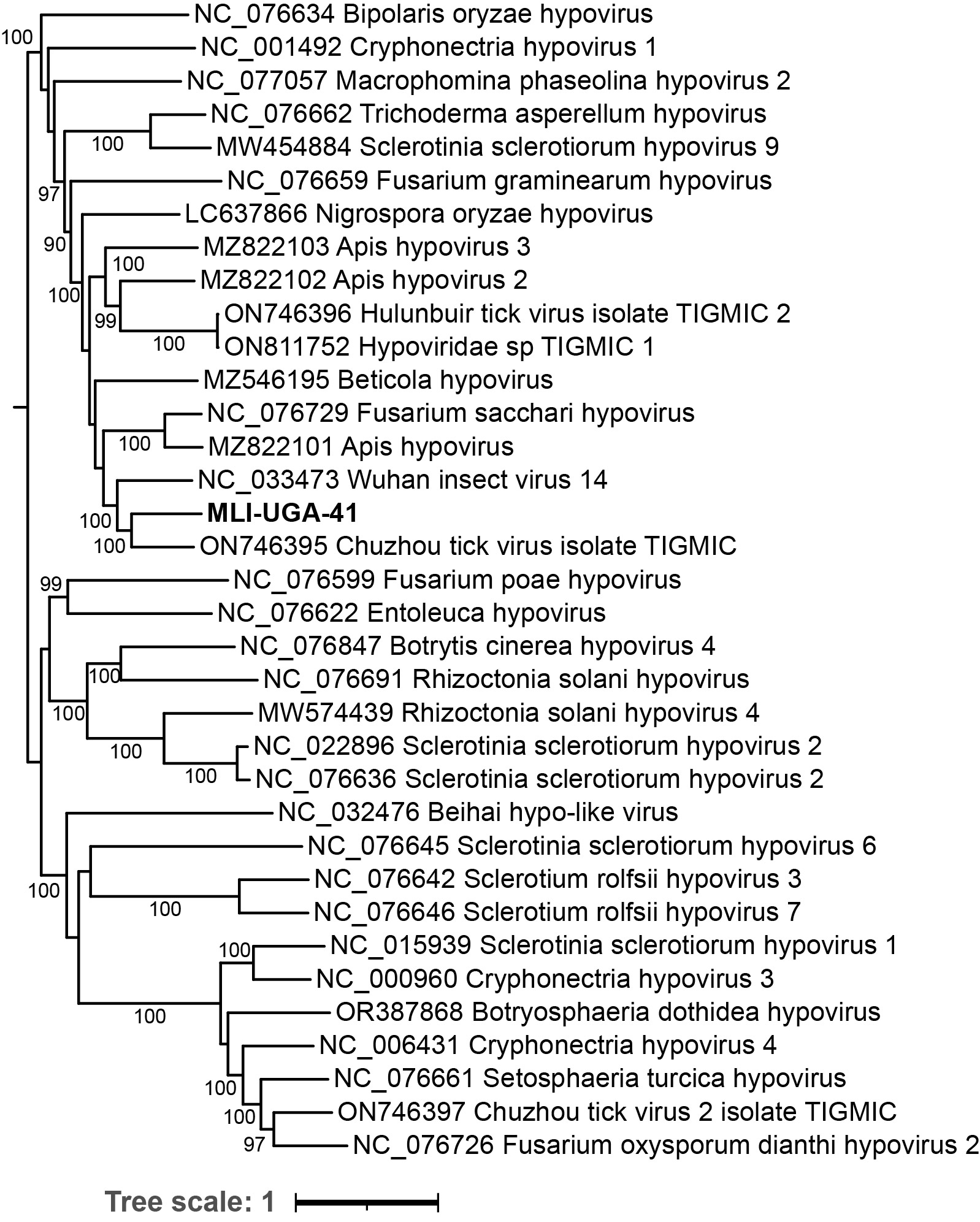

Supplement: Supplementary file 1 — Supplementary material [file mmc1.docx]
